# Effect of Native American ancestry on iron-related phenotypes of Alabama hemochromatosis probands with *HFE *C282Y homozygosity

**DOI:** 10.1186/1471-2350-7-22

**Published:** 2006-03-13

**Authors:** James C Barton, Ellen H Barton, Ronald T Acton

**Affiliations:** 1 Southern Iron Disorders Center, Birmingham, Alabama, USA; 2 Department of Medicine, University of Alabama at Birmingham, Birmingham, Alabama, USA; 3 Immunogenetics Program and Department of Microbiology, University of Alabama at Birmingham, Birmingham, Alabama, USA

## Abstract

**Background:**

In age-matched cohorts of screening study participants recruited from primary care clinics, mean serum transferrin saturation values were significantly lower and mean serum ferritin concentrations were significantly higher in Native Americans than in whites. Twenty-eight percent of 80 Alabama white hemochromatosis probands with *HFE *C282Y homozygosity previously reported having Native American ancestry, but the possible effect of this ancestry on hemochromatosis phenotypes was unknown.

**Methods:**

We compiled observations in these 80 probands and used univariate and multivariate methods to analyze associations of age, sex, Native American ancestry (as a dichotomous variable), report of ethanol consumption (as a dichotomous variable), percentage transferrin saturation and log_e _serum ferritin concentration at diagnosis, quantities of iron removed by phlebotomy to achieve iron depletion, and quantities of excess iron removed by phlebotomy.

**Results:**

In a univariate analysis in which probands were grouped by sex, there were no significant differences in reports of ethanol consumption, transferrin saturation, log_e _serum ferritin concentration, quantities of iron removed to achieve iron depletion, and quantities of excess iron removed by phlebotomy in probands who reported Native American ancestry than in those who did not. In multivariate analyses, transferrin saturation (as a dependent variable) was not significantly associated with any of the available variables, including reports of Native American ancestry and ethanol consumption. The independent variable quantities of excess iron removed by phlebotomy was significantly associated with log_e _serum ferritin used as a dependent variable (p < 0.0001), but not with reports of Native American ancestry or reports of ethanol consumption. Log_e _serum ferritin was the only independent variable significantly associated with quantities of excess iron removed by phlebotomy used as a dependent variable (p < 0.0001) (p < 0.0001; ANOVA of regression).

**Conclusion:**

We conclude that the iron-related phenotypes of hemochromatosis probands with *HFE *C282Y homozygosity are similar in those with and without Native American ancestry reports.

## Background

Hemochromatosis associated with homozygosity for *HFE *C282Y on Ch6p occurs predominantly in whites of western European descent, although the iron-related phenotypes of persons with this type of hemochromatosis are variable. Some C282Y homozygotes, especially those diagnosed in medical care, have serum ferritin >1000 ng/mL, hepatic cirrhosis, and severe iron overload proven by measurement of hepatic iron concentration or phlebotomy to achieve iron depletion [1,2]. Other C282Y homozygotes, particularly those diagnosed in screening programs, have little or no target organ injury or iron overload [3-6]. Mutations in iron-related genes other than *HFE *appear to account for severe iron overload in some C282Y homozygotes [7-9]. However, the overall variability of iron-related phenotypes in C282Y homozygotes remains unexplained by differences in dietary factors [10,11] or mutations in known iron-related genes [12-20].

Results of the HEIRS Study reveal that there are small but significant differences between the iron-related screening phenotypes of Native American and those of white participants recruited from primary care settings who did not report a previous diagnosis of hemochromatosis or iron overload [21]. In age-matched cohorts, mean serum transferrin saturation values were significantly lower and mean serum ferritin concentrations were significantly higher in Native Americans than in whites [21]. In these participants, multiple regression analyses revealed that sex, race/ethnicity, and *HFE *genotype were significant independent determinants of TfSat evaluated as a continuous dependent variable; sex, age, and *HFE *genotype were significant independent determinants of SF evaluated as a continuous dependent variable [21]. In a different study, twenty-eight percent of 80 white hemochromatosis probands with *HFE *C282Y homozygosity diagnosed in medical care in Alabama reported that they had one or more grandparents who were Native Americans [22]. Thus, we evaluated the effect of Native American ancestry on the iron-related hemochromatosis phenotypes of these 80 Alabama hemochromatosis probands [22]. We defined iron-related phenotypes as transferrin saturation at diagnosis, log_e _serum ferritin concentration at diagnosis, and quantities of excess iron removed by phlebotomy.

## Methods

### General criteria for selection of study subjects

The performance of this work was approved by the Institutional Review Boards of Brookwood Medical Center and the University of Alabama at Birmingham. All subjects were adults (≥18 years of age) who resided in central Alabama; each identified himself/herself as white. Hemochromatosis probands with *HFE *C282Y homozygosity were diagnosed in routine medical care during the interval 1992 – 2002, but were otherwise unselected [22]. None of the present probands were identified by general population screening or systematic screening of clinic populations. We tabulated observations in the 80 probands who previously reported evaluable country of ancestry information [22]. We excluded persons of sub-Saharan African or African American descent because 1) *HFE *mutations are uncommon among African Americans in central Alabama [3,23]; and 2) the iron-related phenotypes and genotypes of most African Americans with iron overload are dissimilar to those of white persons with hemochromatosis in Alabama [24,25].

### Diagnosis of hemochromatosis and evaluation of iron overload

A presumptive diagnosis of hemochromatosis was based on demonstration of a phenotype defined by persistently elevated transferrin saturation (>60% in men, >50% in women) [2,26,27]. Evaluation for iron overload and its complications were performed as described elsewhere [2,26,27].

We defined "excess iron removed by phlebotomy" as the difference of iron removed by phlebotomy to achieve iron depletion and the estimated quantity of iron absorbed during the period of phlebotomy to achieve iron depletion. We estimated daily iron absorption in these patients using these assumptions: 1) the age of onset of increased iron absorption was defined as 12 years, because excess iron absorption likely begins in adolescence or early adulthood in C282Y homozygotes [28]; 2) the average patient in the present study had phlebotomy on alternate weeks [29]; 3) daily iron absorption was the same from age 12 years until iron depletion was achieved by phlebotomy; and 4) each year consists of 365 days. These estimates do not include allowances for growth and development, menstrual blood loss, childbirth, lactation, surgical or laboratory blood losses, blood donation, dietary preferences, or other factors.

Food intake recall or other dietary reports were not available in the present cases.

### Determination of ancestry

Questionnaire and interview design to obtain data on countries of ancestry and to permit estimation of frequencies of countries of ancestry reports are described elsewhere [22]. This permitted computation of individual country of ancestry scores which reflect proportional or percentage national ancestry, including Native American ancestry [22].

### Ethanol consumption

We reviewed charts on each of the patients in the present study, and tabulated their self-reports of daily ethanol consumption. We categorized these in this manner: non-drinkers (reported consuming no ethanol); moderate drinkers (reported ethanol consumption estimated at <30 g daily); and heavy drinkers (reported ethanol consumption estimated at ≥30 g daily). Because some proband subgroups based on reports of ethanol consumption were very small, we analyzed these data as dichotomous variables (non-drinkers or drinkers) in multivariate analyses.

### Iron-associated measurements

Serum iron concentration, total serum iron-binding capacity, and serum ferritin concentration were measured at diagnosis using routine automated methods and blood specimens obtained after an overnight fast. Transferrin saturation was expressed as the quotient of serum iron and iron-binding capacity × 100%. In some cases, percutaneous biopsy specimens of liver were obtained as an adjunct to hemochromatosis diagnosis and evaluation of hepatic pathology. Phlebotomy to induce iron depletion was performed as previously described; one unit of phlebotomy was defined as ~500 mL of blood and 200 mg of iron [26]. Iron overload was defined by a) elevation of serum ferritin concentration (≥300 ng/mL in males, ≥200 ng/mL in females) without other explanation; b) 3+ or 4+ intrahepatocytic iron visualized using Perls' staining; c) hepatic iron index ≥1.9; or 4) removal of ≥2.0 g Fe by therapeutic phlebotomy [30]. Iron depletion was defined as complete when the serum ferritin level was 10 – 20 ng/mL [26].

### *HFE *mutation analyses

*HFE *mutation analyses using genomic DNA obtained from buffy coat were performed as described previously [27].

### Statistical considerations

The dataset consisted of observations on 80 evaluable hemochromatosis probands with *HFE *C282Y homozygosity [22]. Observations on age, sex, report or no report of Native American ancestry, transferrin saturation, and serum ferritin at diagnosis, and the units of phlebotomy to achieve iron depletion were available in each proband. A computer spreadsheet (Excel 2000, Microsoft Corp., Redmond, WA) and a statistical program (GB-Stat, v. 8.0 2000, Dynamic Microsystems, Inc., Silver Spring, MD) were used to perform the present analyses. Serum ferritin measurements were normalized by natural logarithmic (log_e_) transformation for analysis [31]. We used separate multiple regression models to examine transferrin saturation, log_e _serum ferritin, and quantities of excess iron removed by phlebotomy as dependent variables (with each as independent variables in models for the other two). Age, transferrin saturation, log_e _serum ferritin, and quantities of excess iron removed by phlebotomy were evaluated as continuous variables; units of phlebotomy to achieve iron depletion was evaluated in univariate analyses only. Sex, Native American ancestry, and reports of ethanol consumption were evaluated as dichotomous variables. Most descriptive data are displayed as enumerations, percentages, mean ± 1 standard deviation (SD), or antilogs of mean log_e_-transformed SF data (95% confidence intervals (95% CI)), or ranges. Age and iron-related phenotype data were rounded to the nearest integer for presentation. Frequency data were compared using Pearson Chi-square tests or Fisher's exact test, as appropriate. Means were compared using Student's t-test (two-tail). Correlation matrices with Bonferroni correction were used to evaluate the relationships of multiple variables. Multiple regression models were also used to evaluate the relationships of the variables age, sex, Native American ancestry, transferrin saturation, log_e _serum ferritin at diagnosis, and units of phlebotomy to achieve iron depletion. Accuracy of the regression models was estimated using analysis of variance (ANOVA). Values of p < 0.05 were defined as significant.

## Results

### General characteristics of hemochromatosis probands

There were reports from 80 evaluable probands (47 men, 33 women) [22]. At diagnosis, their mean age at diagnosis was 51 ± 14 years (range 18 – 78 years). Iron overload was present in 47 men and 31 women. Sixteen percent had hepatic cirrhosis demonstrated by biopsy, 16% had arthropathy typical of hemochromatosis, 19% had diabetes mellitus, and none had cardiomyopathy. Fifteen percent of men had hypogonadotrophic hypogonadism.

The mean ages of men and women were similar (52 ± 15 years vs. 51 ± 12 years; p = 0.2204). Native American ancestry was reported by 10 men (21.3%) and 12 women (36.4%) (p = 0.1368, Chi-square test). Mean transferrin saturation was significantly higher in men than in women (85 ± 13% vs. 77 ± 19%; p = 0.0105). The mean serum ferritin concentration was significantly greater in men than in women (1062 ng/mL (95% CI: 770, 1464) vs. 433 ng/mL (95% CI: 302, 620)) (p = 0.0003). The mean units of phlebotomy required to achieve iron depletion in men was significantly greater than in women (35 ± 33 units vs. 20 ± 21 units; p = 0.0210). The mean quantity of excess iron removed by phlebotomy was significantly greater in men than in women (6534 ± 5578 mg vs. 3750 ± 3744 mg, respectively; p = 0.0162).

### Ethanol consumption

Self-reports of ethanol consumption in the present subjects are displayed in Table 2. The frequencies of non-drinkers and drinkers were similar in men with or without Native American ancestry (p = 0.3659; Fisher's exact test), and in women with or without Native American ancestry (p = 0.3474; Fisher's exact test). However, self-reports of ethanol consumption were significantly greater in all 47 men than in all 33 women (p = 0.0218; Chi-square analysis). Ten men (21.3%) reported that they consumed ≥30 g of ethanol daily; one woman (3.0%) reported she consumed ≥30 g of ethanol daily (p = 0.0180; Fisher's exact test).

### Univariate analyses of phenotypes with and without reports of Native American ancestry

In probands grouped by sex, mean ages at diagnosis, transferrin saturation values, log_e _serum ferritin concentrations, units of phlebotomy to achieve iron depletion, and mean quantities of excess iron removed by phlebotomy were not significantly different in those who reported Native American ancestry and in those who did not report Native American ancestry (Table 1).

**Table 1:** Characteristics of Alabama hemochromatosis probands with *HFE *C282Y homozygosity^1^

Characteristic	Age, y	Transferrin saturation, %	Serum ferritin, ng/mL	Iron removed to achieve iron depletion, mg	Excess iron removed by phlebotomy, mg^3^
Men with report of Native American ancestry (n = 10)^2^	46 ± 10	84 ± 15	1348 (538, 3380)	7250 ± 5082	6827 ± 4701
Men without report of Native American ancestry (n = 37)	53 ± 16	85 ± 12	992 (700, 1404)	6962 ± 6829	6484 ± 5773
Value of p	0.1532	0.8966	0.4988	0.9156	0.8917
					
Women with report of Native American ancestry (n = 12)^2^	48 ± 10	71 ± 20	465 (198, 1093)	5066 ± 5398	4660 ± 4640
Women without report of Native American ancestry (n = 21)	53 ± 13	80 ± 18	415 (288, 598)	3378 ± 3444	3271 ± 3215
Value of p	0.2948	0.2251	0.4194	0.4194	0.4402

^1^Age, transferrin saturation at diagnosis, and units of phlebotomy to achieve iron depletion data are displayed as mean ± 1 standard deviation (SD). Serum ferritin concentrations at diagnosis are displayed as mean (95% confidence intervals) of antilogs of log_e_-transformed measurements. Comparisons of mean variables of respective subgroups of men and women were made using univariate technique (two-tail Student t-test).

^2^The mean proportion of Native American ancestry (± 1 SD) estimated as previously described [22] was 24.3 ± 11.9 in men and 32.3 ± 22.9 in women (p = 0.3119).

^3^These data were computed using quantities of iron removed by phlebotomy to achieve iron depletion, and the assumptions described in Methods, Diagnosis of hemochromatosis and evaluation of iron overload.

**Table 2:** Self-reports of ethanol consumption of Alabama hemochromatosis probands with *HFE *C282Y homozygosity^1^

Characteristic	No ethanol consumption, n (%)	Ethanol consumption, n (%)
Men with report of Native American ancestry (n = 10)	5 (50.0)	5 (50.0)
Men without report of Native American ancestry (n = 37)	14 (37.8)	23 (62.2)
Women with report of Native American ancestry (n = 12)	7 (58.3)	5 (41.7)
Women without report of Native American ancestry (n = 21)	15 (71.4)	6 (28.6)

### Correlation of phenotype data and reports of Native American ancestry

A correlation matrix with Bonferroni correction was used to evaluate relationships of age, sex, report (or no report) of Native American ancestry, report (or no report) of ethanol consumption, transferrin saturation, log_e _serum ferritin concentration, and quantities of excess iron removed by phlebotomy. There was no significant correlation of Native American ancestry reports and other variables. There were significant correlations of log_e _serum ferritin concentration with a) male sex (p < 0.01) and b) quantities of excess iron removed by phlebotomy (p < 0.01).

### Multiple regression analyses of transferrin saturation

Straight regression was used to evaluate percentage transferrin saturation as a dependent variable. The p values associated with the available independent variables were: age, 0.0818; sex, 0.3382; report of Native American ancestry, 0.1082; report of ethanol consumption, 0.8770; log_e _serum ferritin concentration, 0.0520; and quantities of excess iron removed by phlebotomy, 0.3655. ANOVA of this regression yielded p = 0.0675. Forward stepwise regression analysis was performed to determine if any of the available independent phenotype variables were useful in this model. No independent variable was significantly associated with percentage transferrin saturation, including Native American ancestry (p = 0.1082).

### Multiple regression analyses of serum ferritin concentrations

Straight regression was used to evaluate log_e _serum ferritin as a dependent variable. The p values associated with the available independent variables were: age at diagnosis, 0.1122; male sex, 0.0608; report of Native American ancestry, 0.2537; report of ethanol consumption, 0.4959; serum transferrin saturation, 0.0520; and quantities of excess iron removed by phlebotomy, <0.0001. ANOVA of this regression yielded p < 0.0001. In forward stepwise regression analyses, the only independent variable significantly associated with log_e _serum ferritin was quantities of excess iron removed by phlebotomy (p < 0.0001).

### Multiple regression analyses of quantities of excess iron removed by phlebotomy

Straight regression was used to evaluate quantities of excess iron removed by phlebotomy as a dependent variable. Forward stepwise regression analysis was used to determine which of the available independent phenotype variables were useful in this model. Regardless of the addition of other independent variables to the model, log_e _serum ferritin was the only independent variable significantly associated with quantities of excess iron removed by phlebotomy (p < 0.0001 for all combinations of the available independent variables) (p < 0.0001, ANOVA of regression, for all combinations of the available independent variables).

## Discussion

Native American ancestry was not associated with significantly lower mean transferrin saturation percentages in hemochromatosis probands with *HFE *C282Y homozygosity than in probands without Native American ancestry. The multivariate model did not reveal a significant statistical association of transferrin saturation with Native American ancestry or other available independent variables. In the HEIRS Study, mean values of transferrin saturation were significantly lower in participants grouped by sex who reported that they were Native Americans than in whites, although the absolute differences in the mean transferrin saturation values between these two race/ethnicity groups were small [21]. There were no significant differences in mean transferrin saturation values in Native Americans and whites grouped by sex who had neither *HFE *C282Y nor *HFE *H63D [21].

Native American ancestry was not associated with significantly greater log_e _serum ferritin concentration in hemochromatosis probands with *HFE *C282Y homozygosity than we observed in probands without Native American ancestry reports. The multivariate model did not reveal a significant statistical association of log serum ferritin concentration with Native American ancestry or other available independent variables. In the HEIRS Study, mean values of log_e _serum ferritin concentration were significantly higher in participants grouped by sex who reported that they were Native Americans than in whites, although these absolute differences were also small [21]. There were no significant differences in mean serum ferritin values in Native Americans and whites grouped by sex who had neither C282Y nor H63D [21].

Quantity of excess iron removed by phlebotomy was the only significant independent variable associated with log_e _serum ferritin concentration in the present study. This is consistent with previous analyses that reveal significant positive relationships of units of phlebotomy or quantities of iron removed to achieve iron depletion in *HFE *C282Y homozygotes [32,33]. Although elevated serum ferritin concentrations sometimes occur in association with or as a consequence of ethanol consumption [34,35], we observed no significant association of log_e _serum ferritin concentration (or quantities of excess iron removed by phlebotomy) with reports of ethanol consumption in the present study.

There are uncertainties in the present study. The overall number of probands available for analysis was small, and thus evaluation of greater numbers of probands may have revealed significant differences not detected herein. The proportional Native American ancestry reported by some of the present probands was low [22]. This could explain the similarity of the iron-related phenotypes in probands with or without Native American ancestry reports. The Native American ancestry reported by the present probands was predominantly Cherokee or Creek heritage [22], consistent with accounts of early Alabama history [36-39] and with U.S. Census data on Alabama since 1820 [40-42]. However, there are no reports of the effect of ancestry derived in part from subgroups of Native Americans typical of regions other than Alabama on iron-related phenotypes in *HFE *C282Y homozygotes. We did not examine effects of Native American ancestry on possible non-biochemical expression of hemochromatosis and iron overload in the present subjects. Although 98% of the present subjects had iron overload, the proportions of these patients who had hepatic cirrhosis demonstrated by biopsy, arthropathy typical of hemochromatosis, diabetes mellitus, cardiomyopathy, or hypogonadotrophic hypogonadism were low. In addition, the occurrence of these complications of iron overload in persons with hemochromatosis is, in general, related to the severity of iron overload [43]. However, we observed no significant differences in quantities of excess iron removed by phlebotomy as a function of race/ethnicity in the present analyses. Thus, it is unlikely that there were significant differences in clinical complications of iron overload in the present patients that could be attributed to reports of Native American ancestry, although this is unproven. Iron-related phenotypes are also influenced by common liver disorders [34,35,44-47], body mass index [48,49], and rates of menstrual blood loss [50], although most of the subjects evaluated in these reports were not Native Americans. Further, evaluation of these variables was beyond the scope of the present study.

## Conclusion

We conclude that the iron-related phenotypes of hemochromatosis probands with *HFE *C282Y homozygosity are similar in those with and without Native American ancestry reports. The present results suggest that the occurrence of Native American ancestry in whites would not significantly affect outcomes of hemochromatosis diagnosis and screening with transferrin saturation or serum ferritin measurements. This is consistent with previous observations that the percentage of Native American ancestry reports and the proportional Native American ancestry of the present probands were similar to those of white control subjects who resided in central Alabama [22]. The transferrin saturation phenotype screening of two Native American populations in Canada (1407 "Native Americans," 310 Inuit) revealed no subject with evidence of iron overload [51]. This is consistent with HEIRS Study reports that the frequency of the C282Y allele is low in Native Americans [3,21], and that none of 645 HEIRS Study participants who reported that they were Native Americans had *HFE *C282Y homozygosity [21].

## Competing interests

The author(s) declare that they have no competing interests.

## Authors' contributions

JCB conceived and designed the study, diagnosed and treated the hemochromatosis probands and compiled their clinical data, performed some of the statistical analyses, and contributed to writing the manuscript. EHB compiled country of ancestry data, performed some of the statistical analyses, and contributed to writing the manuscript. RTA compiled data on hemochromatosis probands, performed *HFE *typing of many of the probands, and contributed to writing the manuscript. All authors approved of the manuscript in its final form.

## Pre-publication history

The pre-publication history for this paper can be accessed here:


